# Hsa_circ_0012152 and Hsa_circ_0001857 Accurately Discriminate Acute Lymphoblastic Leukemia From Acute Myeloid Leukemia

**DOI:** 10.3389/fonc.2020.01655

**Published:** 2020-09-02

**Authors:** Shanshan Guo, Bixia Li, Ying Chen, Duobing Zou, Shujun Yang, Yi Zhang, Ningning Wu, Lixia Sheng, He Huang, Guifang Ouyang, Qitian Mu

**Affiliations:** ^1^Ningbo Hospital, School of Medicine, Zhejiang University, Ningbo, China; ^2^School of Medicine, Ningbo University, Ningbo, China; ^3^Laboratory of Stem Cell Transplantation, Ningbo Hospital, School of Medicine, Zhejiang University, Ningbo, China; ^4^Department of Hematology, Ningbo Hospital, School of Medicine, Zhejiang University, Ningbo, China; ^5^Bone Marrow Transplantation Center, The First Affiliated Hospital, School of Medicine, Zhejiang University, Hangzhou, China

**Keywords:** acute lymphoblastic leukemia, acute myeloid leukemia, biomarker, bioinformatics, circRNA

## Abstract

Acute leukemia (AL) is a group of highly heterogeneous hematological malignancies. Circular RNAs (circRNAs) are covalently closed circRNA molecules implicated in the development of many diseases. However, the role of circRNAs in AL remains largely unknown. Therefore, this study aimed to identify new classification diagnostic biomarkers for subgroups of AL. The circRNA expression signatures discriminating acute lymphoblastic leukemia (ALL) from acute myeloid leukemia (AML) were identified by microarray, followed by reverse transcription quantitative polymerase chain reaction (RT-qPCR) validation. Receiver operating characteristic curve analysis was used to evaluate the diagnostic efficiencies of hsa_circ_0001857 and hsa_circ_0012152, and hsa_circ_0012152 was selected for Gene Ontology and Kyoto Encyclopedia of Genes and Genomes analysis. The results showed that the circRNA expression profiles, hsa_circ_0001857, and hsa_circ_0012152 could clearly discriminate ALL from AML. The target genes of hsa_circ_0012152 might be involved in biological processes, such as myeloid cell differentiation, covalent chromatin modification, histone modification, and rat sarcoma (Ras) protein signal transduction, and participate in pathways such as mitogen-activated protein kinase (MAPK) and phosphatidylinositol 3′-kinase (PI3K)-Akt signaling pathway. Hsa_circ_0012152 might be involved in the initiation and development of AML through miR-491-5p/epidermal growth factor receptor (EGFR)/MAPK1 or miR-512-3p/EGFR/MAPK1 axis. Our results showed that circRNA expression profiles and specifically expressed circRNAs were promising classification biomarkers to designate AL into ALL or AML.

## Introduction

Acute leukemia (AL) is a diverse group of hematological malignancies, characterized by aberrant proliferation and accumulation of malignantly transformed hematopoietic stem cells in the bone marrow and other hematopoietic tissues (1). Acute leukemia was separated into two distinct entities, acute lymphoblastic leukemia (ALL), and acute myeloid leukemia (AML), according to their diverse morphologies, prognoses, and preferred treatment protocols (2). Accurate and timely diagnosis is critical for the clinical management of acute leukemia, as the prognosis and preferred treatment options for ALL and AML vary considerably (1). Although methods such as morphology and immunohistochemistry can be used to distinguish ALL from AML (3, 4), conventional methods require a high grade of expertise and have no implications for pathogenesis, prognosis, and treatment. Later, immunology, cytogenetics, and molecular biology were found to be useful in the diagnosis and classification of AL and to provide information for understanding the pathogenesis, assessing prognosis, and selecting treatment protocols (5). However, these methods still had disadvantages: they had different laboratory standards, they were time consuming and expensive, and no single test could accurately discriminate ALL from AML alone.

Circular RNAs (circRNAs) are a class of covalently closed single-stranded RNA molecules whose 3′ and 5′ ends are linked by a back-splice event (6). Some of them have been proven to be structurally stable (7), tissue and cell specific (8), abundantly expressed in various tissues and cells (9), and evolutionarily conserved (10). Emerging evidence has shown that some circRNAs are involved in many biological processes and a variety of human diseases, including innate immunity (11), inflammation (12), tumorigenesis (13, 14), aging (15), diabetes mellitus (16), neurological disorders (17), and cardiovascular diseases (18) by acting as microRNA (miRNA) sponges (19–21), regulating splicing and transcription (22, 23), interacting with RNA-binding proteins (24, 25), or serving as protein translation templates (26–28). These findings indicated that circRNAs were promising biomarkers and therapeutic targets for some human diseases. Increasing evidence showed that some circRNAs were potential diagnostic and prognostic biomarkers and might be involved in the initiation and development of some hematological malignancies. For example, f-circPR (14), f-circM9 (14), and circAF4 (29) were specifically expressed in leukemia cell lines and patients with *PML-RAR*α, *MLL-AF9*, and *MLL-AF4*, respectively, and might be involved in the initiation and development of AL. Therefore, they were potential biomarkers and therapeutic targets for acute leukemia. CircMYBL2 (30), which was upregulated in AML with Fms-like tyrosine kinase-3 internal tandem duplication (*FLT3-ITD*) mutations, could enhance the translational efficiency of FLT3 kinase by recruiting PTBP1 to promote the development and confer drug resistance of *FLT3-ITD*-positive AML. Circ-RPL15 (31), which was upregulated in patients with chronic lymphoid leukemia (CLL) compared with healthy individuals, could serve as a diagnostic biomarker for CLL with the area under the curve (AUC) of 0.84 and was always associated with a poor outcome. It contributed to the progression of CLL by acting as a sponge of miR-146b/3p to release *RAF1*. However, whether circRNA expression profiles and certain specifically expressed circRNAs could be used as classification biomarkers to discriminate ALL from AML was unclear.

In this study, the circRNA expression profiles of AML, ALL, and healthy individuals were found to be markedly different. Hsa_circ_0012152 and hsa_circ_0001857, upregulated in AML and ALL, respectively, could clearly discriminate ALL from AML, and their combination could improve the diagnostic efficiency. Hsa_circ_0012152 might be involved in the progression of AML through miR-491-5p/epidermal growth factor receptor (EGFR)/MAPK1 or miR-512-3p/EGFR/MAPK1 axis.

## Materials and Methods

### Patients and Specimen Collection

Bone marrow (BM) samples from 49 newly diagnosed patients with AML, 43 newly diagnosed patients with ALL, and 8 healthy individuals were used in this study. Five AML samples, three ALL samples, and three healthy individual samples were used for the microarray analysis of circRNA expression profiles, and the other 89 samples were used for RT-qPCR amplification assay. Mononuclear cells from BM aspirates were separated by Ficoll–Paque Plus (GE Healthcare, Uppsala, Sweden) density gradient centrifugation. Written informed consents were obtained from all participants involved in this study before using the clinical samples for research purpose. The protocol of this study was approved by the ethics committee of the Ningbo Hospital of Zhejiang University and carried out in accordance with the Declaration of Helsinki. The diagnoses of AML and ALL were made in accordance with the 2016 World Health Organization Classification of Tumors of Haematopoietic and Lymphoid Tissues (2).

### CircRNA Microarray

Total RNAs were quantified using a NanoDrop ND-1000 spectrophotometer (Thermo Scientific, Wilmington, DE, United States). The sample preparation and microarray hybridization were performed based on the Arraystar’s standard protocols by Shanghai KangCheng Bio-tech Co. Ltd. Total RNAs were digested with RNase R (Epicenter, Inc., Madison, WI, United States) to remove linear RNAs and enrich circRNAs. Then, enriched circRNAs were amplified and transcribed into fluorescent complementary RNA (cRNA) by a random priming method (Arraystar Super RNA Labeling Kit; Arraystar, Rockville, MD, United States). The labeled cRNAs were hybridized onto an Arraystar Human circRNA Array V2 (8 × 15K, Arraystar, Rockville, MD, United States). After washing the slides, the arrays were scanned using an Agilent Scanner G2505C.

### Microarray Data Analysis

Agilent Feature Extraction software (version 11.0.1.1, Agilent Technologies) was used to analyze acquired circRNA array images. Quantile normalization and differentially expressed circRNAs were performed by R software (Version 3.6.6, (32)). Differentially expressed circRNAs with statistical significance between two groups were identified with | log_2_FC| ≥ 2 and false discovery rate (FDR) <0.05 filtering. Differentially expressed circRNAs with statistical significance were presented with a heatmap and volcano plot using heatmap R package and ggplot R package. Hierarchical cluster analysis (33) was performed using R software to display the distinguishable circRNA expression profiles among samples.

### Total RNA Extraction and RT-qPCR

Total RNA was extracted with TRIzol reagent (Takara Bio Inc., Japan) following the manufacturer’s protocols. The purity and concentration of RNA samples were detected with a NanoDrop ND-1000 spectrophotometer (Thermo Scientific, Wilmington, DE, United States). The cDNAs were synthesized using a PrimeScript RT Master Mix (Takara Bio Inc., Japan) following the manufacturer’s protocol. The RT-qPCR was performed in the Stepone Real-Time PCR System (Applied Biosystems, Life Technologies, Carlsbad, CA, United States) using an SYBR Green PCR kit (Takara Bio Inc., Japan). Glyceraldehyde 3-phosphate dehydrogenase (GAPDH) was employed as the endogenous control. A comparative cycle threshold (2^–Δ^ ^Δ^ ^CT^) method was used to analyze the gene expression level, and three technological replicates were used to ensure the reliability of the analysis. The RT-qPCR reaction system with 20 μl of volume consisted of 1 μl of cDNA, 1.6 μl of primer, 10 μl of SYBR Green, 0.4 μl of ROX, and 7 μl of RNase-free H_2_O. The amplification of RT-qPCR reaction was carried out at 95°C for 1 min, followed by 40 cycles at 95°C for 15 s, 64°C for 30 s, and 72°C for 32 s to record fluorescence, and finally followed by the melting program at 95°C for 15 s, 60°C for 1 min, 95°C for 15 s, and 60°C for 15 s. The Primer sequences are shown in Supplementary Table S1.

### Construction of Competing Endogenous RNA Network

The target miRNA and miRNA target genes of hsa_circ_0012152 were collected from starBase 3.0^1^. A protein–protein interaction (PPI) network was established using the STRING database^2^ and presented as a graph using Cytoscape software (Version 3.6.1). Hub proteins were the proteins having an extremely high connection with other proteins in the PPI network and were vital in many biological processes (34, 35). They were identified using the Degree algorithm plug-in of Cytoscape software. A circRNA–miRNA–mRNA crosstalk network and competing endogenous RNA (ceRNA) network were also built using Cytoscape software.

### Gene Ontology and Kyoto Encyclopedia of Genes and Genomes Pathway Analyses for Target Genes

Gene Ontology (GO) is a gene function classification system widely used in the field of bioinformatics. It simply annotates genes from the cellular component, molecular function, and biological process. Kyoto Encyclopedia of Genes and Genome (KEGG)^3^ is a reference knowledge base for the biological interpretation of genome sequences and high-throughput data. GO and KEGG pathway analysis was performed using the clusterProfile R package, and the results were presented as graphs using the ggplot and GOplot R package.

### Statistical Analysis

All data were presented as the median or mean ± standard error of the mean (SEM) and analyzed using SPSS 23.0 software or GraphPad Prism 7.0 software with a two-tailed *P* value. Analysis of variance, Student *t* test, or Kruskal–Wallis *H* tests were used as appropriate to determine the differences between groups. Receiver operating characteristic (ROC) curves were used to evaluate the diagnostic efficiencies of different circRNAs. Youden’s index was used to identify the best cutoff value and calculate the specificity, sensitivity, positive predictive value, and negative predictive value. The diagnostic efficiency for the combination of biomarkers was assessed using serial test, parallel test, or calculating weight coefficients for every biomarker, obtaining the largest possible AUC in ROC analysis. Weight coefficients were valuated using a multivariable-adjusted logistic regression model. A *P* < 0.05 was considered statistically significant.

## Results

### CircRNA Expression Profiles Distinguished ALL From AML

**FIGURE 1:**
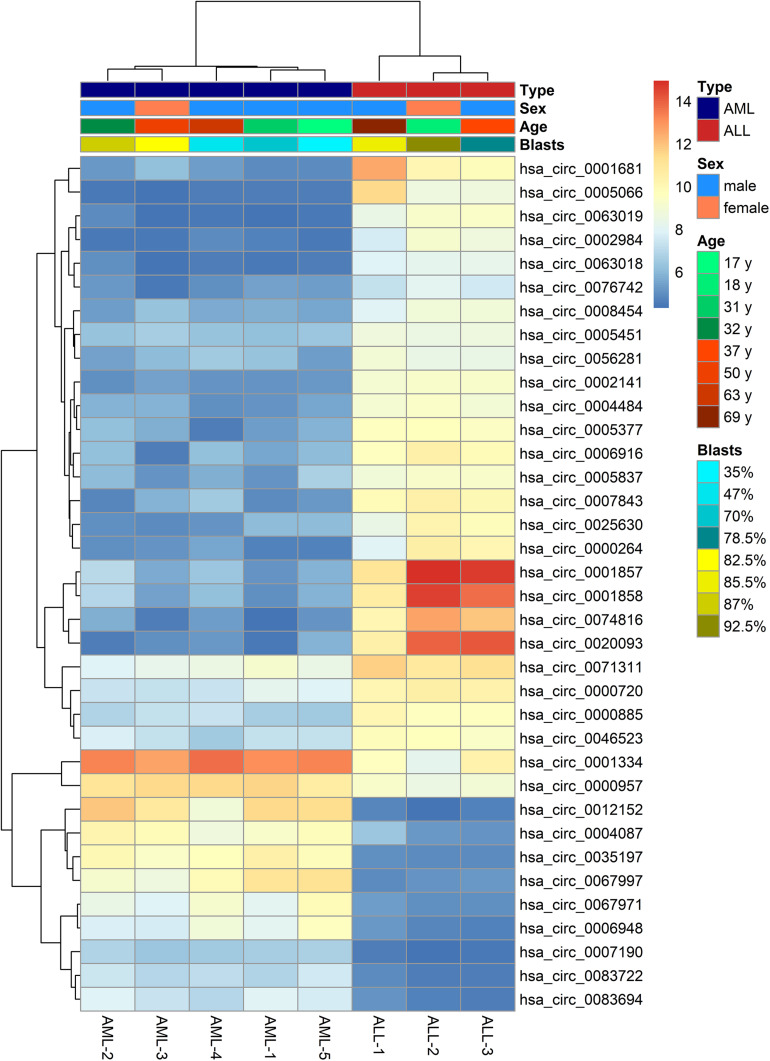
Heatmap of differentially expressed circular RNAs (circRNAs) between acute lymphoblastic leukemia (ALL) and acute myeloid leukemia (AML). The horizontal axis shows the samples, and the vertical axis shows differentially expressed circRNAs. That is, there is one circRNA for each line, and one sample for each column. Red and gray indicate upregulated and downregulated expression, respectively, in ALL compared with AML; the darker the color, the greater the difference.

**FIGURE 2:**
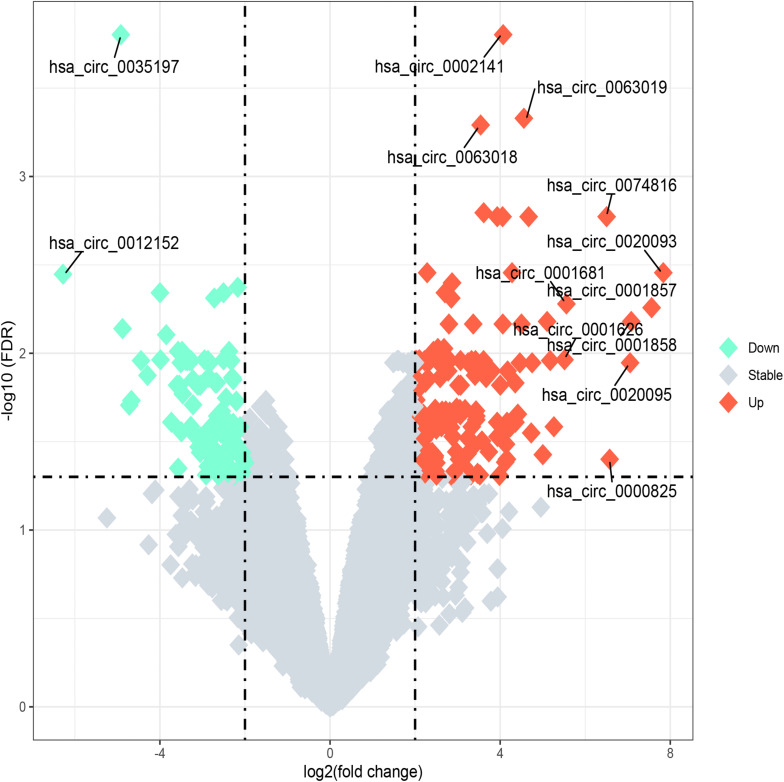
Volcano plot of differentially expressed circular RNAs (circRNAs) between acute lymphoblastic leukemia (ALL) and acute myeloid leukemia (AML). “Log_2_ (fold change)” is plotted on the horizontal axis, and “-log_10_ (FDR)” is plotted on the vertical axis; that is, the larger the “|log_2_ (fold change)|,” the greater the difference, and the larger the “-log_10_ (FDR),” the more significant the statistical difference. Tomato and aquamarine, respectively, represent the upregulated and downregulated circRNAs with |log_2_FC| ≥ 2 and FDR < 0.05, while the gray represents the circRNAs with |log_2_FC| < 2 or FDR ≥ 0.05.

The microarray analysis of circRNA expression profiles was performed on 11 BM samples, including 3 from patients with ALL, 5 from patients with AML, and 3 from healthy individuals. The circRNA expression profiles of AML and ALL were chosen for further analysis to identify circRNAs differentially expressed between ALL and AML. The results showed that 36 circRNAs were differentially expressed between ALL and AML with a threshold of |log_2_FC| > 2 and FDR < 0.05, including 25 upregulated circRNAs and 11 downregulated circRNAs in ALL compared with AML (Figures 1, 2). An unsupervised two-way, hierarchical cluster analysis (33) was performed on the circRNA expression profiles of the eight samples. As shown in Figure 1, the eight samples grouped into two clusters based on their disease type rather than age, sex, or percentage of blasts. The five AML samples were grouped as a cluster, and the three ALL samples were grouped as another cluster, which reflected that these differentially expressed circRNAs might be intrinsic regulators for the initiation of ALL and AML.

### Expression Levels of Hsa_circ_0012152 and Hsa_circ_0001857 in a Cohort of Patients With AL

**FIGURE 3:**
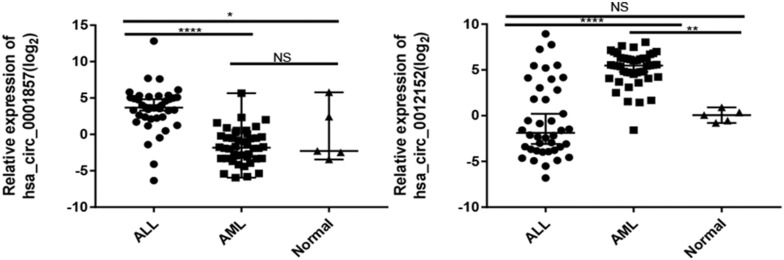
Expression of hsa_circ_0001857 and hsa_circ_001252 in a cohort of patients with acute leukemia. *P < 0.05, **P < 0.01, ****P < 0.0001; NS, no significance.

The expression levels of five circRNAs (hsa_circ_0001857, hsa_circ_0001858, hsa_circ_0074816, hsa_circ_0001681, and hsa_circ_0012152) that were chosen from the top 10 differentially expressed circRNAs between ALL and AML were quantified by RT-qPCR using the same cohort of patients as the circRNA microarray to validate the results of circRNA microarray and identify potential biomarkers to distinguish ALL from AML (Supplementary Figure S1; the information about the top 10 differentially expressed circRNAs between ALL and AML is shown in Supplementary Table S2). Their expression patterns were consistent with the results of the circRNA microarray, indicating high reliability of the circRNA microarray and the potential of these differentially expressed circRNAs as biomarkers to discriminate ALL from AML. Then, the expression levels of hsa_circ_0001857 and hsa_circ_0012152 in 40 newly diagnosed patients with ALL, 44 newly diagnosed patients with AML, and 5 healthy individuals were quantified by RT-qPCR (Figure 3) for more significant differences in their expression between ALL and AML. The results showed that hsa_circ_0001857 was markedly upregulated in ALL compared with AML (12.73 vs 0.28, *P* < 0.0001) or healthy individuals (12.73 vs 0.24, *P* < 0.05), and hsa_circ_0012152 was markedly upregulated in AML compared with ALL (44.63 vs 0.27, *P* < 0.0001) or healthy individuals (44.63 vs 1.04, *P* < 0.0001). These results indicated that hsa_circ_0001857 and hsa_circ_0012152 were potential classification biomarkers to discriminate ALL from AML due to their differential expression patterns between ALL and AML. The association between hsa_circ_0012152 and hsa_circ_0001857 with several clinical features is shown in Table 1. The expression of hsa_circ_0012152 in AML was independent of age, sex, as well as *AML1-ETO*, *PML-RAR*α, *FLT3-ITD*, *NPM*, and other known driver genes. The expression of hsa_circ_0001857 in ALL was associated with age, and younger patients had lower expression than older patients. However, the expression of hsa_circ_0001857 was independent of sex or known mutations or fusion genes such as *IKZF1* and BCR-ABL. These suggested that hsa_circ_0012152 and hsa_circ_0001857 functioned independently of these commonly known driver genes.

**TABLE 1:** Expression of hsa_circ_0012152 and hsa_circ_0001857 in subtypes of acute myeloid leukemia (AML) and acute lymphoblastic leukemia (ALL).

Features		No.	hsa_circ_0012152	Features		No.	hsa_circ_0001857
			**(ΔCT median or mean ± SEM)^a^**				**(ΔCT median)**

	–	38	4.319	Ph(BCR-ABL)	–	22	7.32
AML1-ETO	+	6	4.666		+	11	7.786
	*P* value		0.5816		*P* value		0.4859
	–	36	4.366	*IKZF1*	–	21	6.7
PML-RARα	+	8	4.391		+	7	7.786
	*P* value		0.3603		*P* value		0.1839
	–	34	4.526 ± 0.2501	MLL	–	34	7.765
FLT3-ITD^a^	+	5	4.573 ± 0.94	rearrangement	+	–	–
	*P* value		0.9639		*P* value		–^b^
	–	34	4.518 ± 0.2688	Subtype	B-ALL	38	7.779
NPM^a^	+	5	4.627 ± 0.5808		T-ALL	–	–
	*P* value		0.8711		*P* value		–^b^
	<51.5 years	22	4.44	Age	<34 years	18	8.108
Age	≥51.5 years	22	4.079		≥34 years	22	6.747
	*P* value		0.3824		*P* value		0.0117*
	Male	25	4.431	Sex	Male	26	7.795
Sex	Female	19	3.857		Female	14	7.772
	*P* value		0.5573		*P* value		0.9888

*No., number. ^*a*^Data of FLT3-ITD and NPM showed a normal distribution. Student’s *t* tests were performed, and the results were represented as mean ± SEM. The remaining data were analyzed using Kruskal–Wallis *H* tests after confirming that the data showed a non-normal distribution, and the results were represented as a median. ^*b*^Positive rate was too low, and no statistical analysis was performed.*

### Hsa_circ_001252 and Hsa_circ_0001857 Could Accurately Discriminate ALL From AML

**FIGURE 4:**
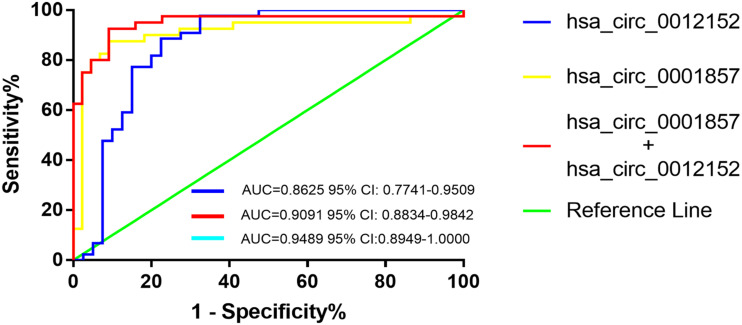
The receiver operating characteristic (ROC) of hsa_circ_00012152 and hsa_circ_0001857 as classification biomarkers to discriminate acute lymphoblastic leukemia (ALL) from acute myeloid leukemia (AML).

Receiver operating characteristic analysis was established to evaluate the efficiency of hsa_circ_0012152 and hsa_circ_00001857 as diagnostic (Supplementary Table S3) or classification (Table 2) biomarkers. The ROC analysis showed that hsa_circ_0012152 could discriminate AML from healthy individuals with a sensitivity of 0.977, a specificity of 1.000, and an AUC of 0.9773 (95% CI, 0.9332–1.0000, *P* = 0.0005; Supplementary Figure S2A), but hsa_circ_0001857 could not discriminate ALL from healthy individuals (AUC = 0.7350, *P* = 0.0896, Supplementary Figure S2B). Hsa_circ_0012152 could discriminate ALL from AML with an AUC of 0.8625 (95% CI, 0.7741–0.9509, *P* < 0.0001), a sensitivity of 0.775, and a specificity of 0.886 (Table 2 and Figure 4). Hsa_circ_0001857 could discriminate ALL from AML with an AUC of 0.9091 (95% CI, 0.8834–0.9842, *P* < 0.0001), a sensitivity of 0.875, and a specificity of 0.909 (Table 2 and Figure 4).

**TABLE 2:** Diagnostic efficiency of hsa_circ_0012512 and hsa_circ_0001857 as classification biomarkers to discriminate acute lymphoblastic leukemia (ALL) from acute myeloid leukemia (AML).

CircRNA	Sensitivity	Specificity	PPV	NPV	AUC	95%CI	*P* value
hsa_circ_0001857	0.875	0.909	0.897	0.888	0.9091	0.883–0.9842	<0.0001
hsa_circ_0012152	0.775	0.886	0.841	0.812	0.8625	0.7741–0.9509	<0.0001
Logistic (hsa_circ_0001857 + hsa_circ_0012152)	0.925	0.909	0.829	0.930	0.9489	0.8596–1.0000	<0.0001
Serial test	0.700	0.977	0.966	0.782	–	–	–
Parallel test	0.950	0.818	0.826	0.947	–	–	–

*Abbreviations: AUC, area under curve; 95% CI, 95% confidence interval; NPV, negative predict value; and PPV, positive predict value.*

Serial and parallel tests were used to improve the efficiency of classification diagnosis to discriminate ALL from AML (Table 2 and Figure 4). The specificity and positive predictive value increased to 0.977 and 0.966, but the sensitivity and negative predictive value decreased to 0.700 and 0.782, respectively, using the serial test. The sensitivity and negative predictive value increased to 0.950 and 0.947, but the specificity and positive predictive value decreased to 0.818 and 0.826, respectively, using the parallel test. Hence, the serial and parallel tests decreased the sensitivity and specificity, respectively. Therefore, a logistic regression predictive model that integrated two or more biomarkers into one biomarker was used, which could comprehensively improve and evaluate the diagnostic efficiency (36, 37). The result of the predictive model suggested that the AUC improved to 0.9489 (95% CI, 0.8596–1.0000, and *P* < 0.0001), the sensitivity increased to 0.925, and the specificity (0.909) reached the optimal level when hsa_circ_0012152 and hsa_circ_0001857 were diagnosed alone. These results indicated that hsa_circ_0012152 and hsa_circ_0001857 were promising potential classification biomarkers to distinguish ALL from AML, and their combination could better identify ALL from AML, although they showed an excellent performance in distinguishing ALL for AML when they were alone. Clinicians expect a clear separation of ALL from AML in the management of AL. That is, the biomarker was expected to have an excellent performance in specificity. Hence, the serial test and logistic regression predictive model might be a better choice.

### Construction of ceRNA Network About hsa_circ_0012152

**FIGURE 5:**
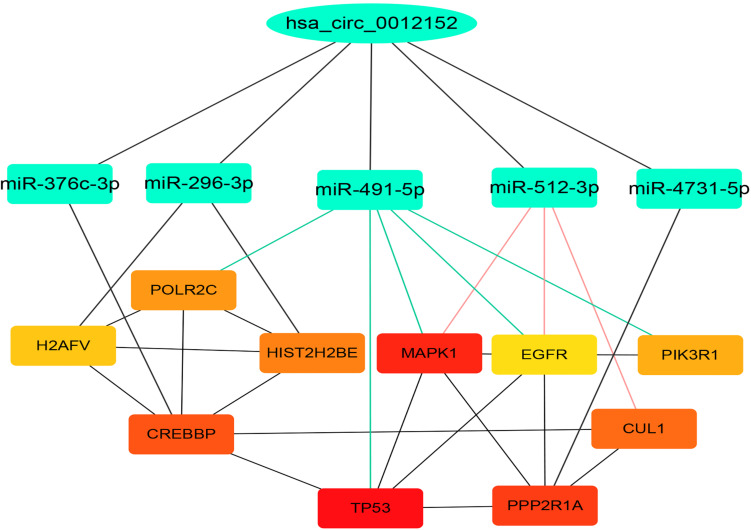
Competing endogenous RNA (ceRNA) network indicating the potential functioning mechanism of hsa_circ_0012152. The top layer shows hsa_circ_0012152, the middle layer shows the five target microRNAs (miRNAs) of hsa_circ_0012152, and the bottom layer shows the target gens of miRNA. The lines between the boxes represent the interaction relationship.

Hsa_circ_0012152 discriminated patients with AML from healthy individuals, or ALL from AML, but hsa_circ_0001857 discriminated only ALL from AML, not patients with ALL from healthy individuals. Hsa_circ_0012152 might be involved in the pathogenesis of AML. Thus, hsa_circ_0012152 was selected for further study. A total of 11 miRNAs were predicted to bind to hsa_circ_0012152 using starBase 3.0, including miR-296-3p, miR-4731-5p, miR-512-3p, miR-5094, miR-451a, miR-376c-3p, miR-3200-3p, miR-491-5p, miR-3150a-3p, miR-6763-5p, and miR-3200-5p. A total of 1,862 target genes were predicted for target genes of the other 10 miRNAs using starBase 3.0, except that miR-6763-5p had no target genes. A PPI network (Supplementary Figure S3) about the 1,862 target genes was established using STRING, and 10 hub genes in the PPI network were identified by the Degree algorithm plug-in of Cytoscape software, namely *TP53*, *MAPK1*, *PPP2R1A*, *CREBBP*, *CUL1*, *HIST2H2BE*, *POLR2C*, *PIK3R1*, *H2AFV*, and *EGFR* (Supplementary Figure S4; the crosstalk network of circRNA*–*miRNA–mRNA is shown in Supplementary Figure S5). Then, a ceRNA network including hsa_circ_0012152, 10 hub genes, and miRNA binding to them, was constructed (Figure 5). In the ceRNA network, hsa_circ_0012152 shared miRNA response elements (MREs) of miR-491-5p with *EGFR*, *MAPK1*, *PIK3R1*, *TP53*, and *POLR2C* and shared MREs of miR-512-3p with *EGFR*, *MAPK1*, and *CUL1*. Importantly, the ceRNA network showed that hsa_circ_0012152 competed with *EGFR* and *MAPK1* to combine miR-491-5p or miR-512-3p, indicating that hsa_circ_0012152 might involve in AML through miR-491-5p/EGFR/MAPK1 or miR-512-3p/EGFR/MAPK1 axis.

### GO and KEGG Analyses

**FIGURE 6:**
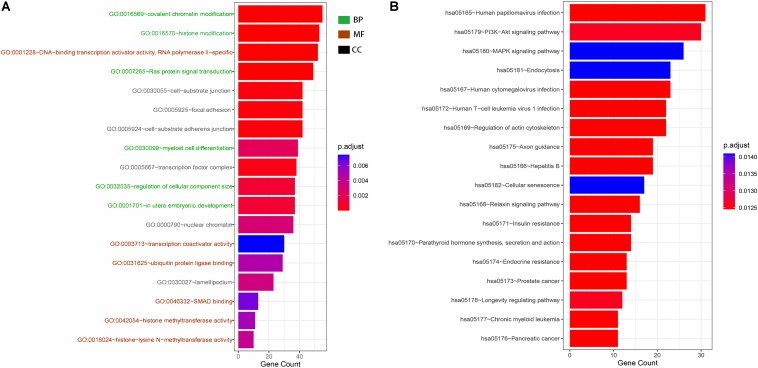
Gene Ontology (GO) and Kyoto Encyclopedia of Genes and Genome (KEGG) functional annotation. **(A)** GO functional annotation of target genes. Green shows the six biological processes (BP) in which target genes are involved, brick red shows the six molecular functions (MF) of the target genes, and black shows the six cellular components (CC) of the target genes. **(B)** KEGG functional annotation of target genes. Each row shows a signaling pathway in which the hsa_circ_0012152 target genes may be involved.

**FIGURE 7:**
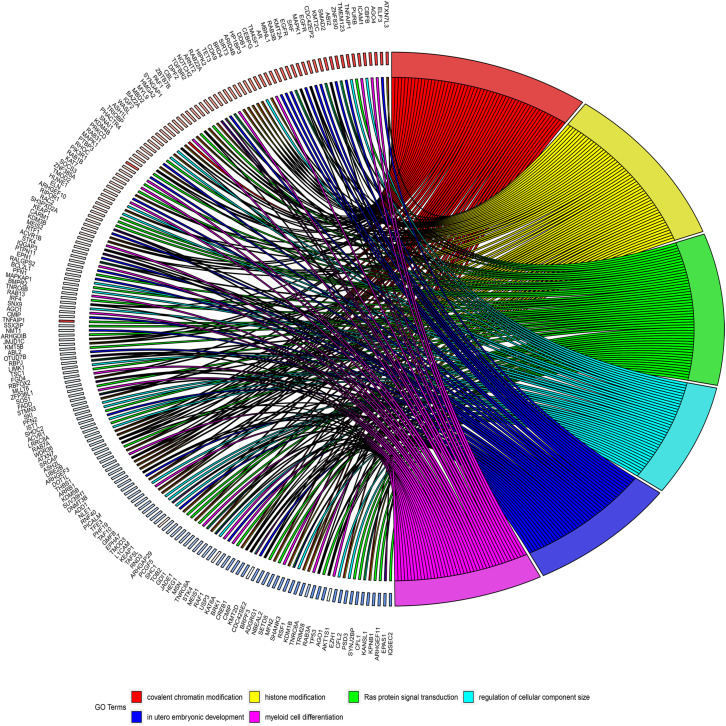
Circos diagrams illustrating six biological processes in which target genes are involved. The right half of the figure shows six biological processes in which the target genes may be involved, including myeloid cell differentiation, and the left half of the figure shows the target genes involved in the six biological processes.

GO and KEGG analyses were used to annotate the functions of the target genes of miR-491-5p and miR-512-3p, as they were promising potential target miRNAs of hsa_circ_0012152. The results of GO analysis (Figures 6A, 7) showed that the target genes might play a role in some biological processes such as “covalent chromatin modification,” “histone modification,” “Ras protein signal transduction,” “regulation of cellular component size,” and “myeloid cell differentiation” and might function as “DNA-binding transcription activator activity, RNA polymerase II-specific,” “histone methyltransferase activity,” and so on. The KEGG analysis (Figure 6B) showed that the target genes might participate in pathways such as “human papillomavirus infection,” “hepatitis B,” “PI3K-Ak*t* signaling pathway,” “MAPK signaling pathway,” “chronic myeloid leukemia,” and so forth.

## Discussion

Currently, the methods of morphology, immunology, cytogenetics, and molecular biology can be used to identify ALL from AML, but these methods still have some disadvantages. They required a high grade of expertise and different laboratory standards, they were time consuming and expensive, and no single test could accurately discriminate ALL from AML alone. MiRNA (38, 39) and long noncoding RNA (lncRNA) (40, 41) have also been shown in recent years to be useful in the diagnosis of AL. However, the role of circRNAs in AL remains largely unknown.

Hence, the initial purpose of this study was to identify new classification biomarkers to distinguish ALL from AML and explore the potential mechanism of circRNAs in AL. The results indicated that circRNA expression profiles could clearly distinguish ALL from AML. The hsa_circ_0001857 was upregulated in patients with ALL compared with AML or healthy individuals and was a promising classification biomarker to discriminate ALL from AML (AUC = 0.9091, *P* < 0.0001). Although hsa_circ_0001857 was differentially expressed between patients with ALL and healthy individuals, it could not discriminate ALL from healthy individuals (AUC = 0.7350, *P* = 0.0896), which might be due to the small sample size. Hsa_circ_0012152 was upregulated in patients with AML compared with ALL or healthy individuals, could discriminate patients with AML from healthy individuals (AUC = 0.9773, *P* = 0.0005), and was a promising potential classification biomarker to discriminate ALL from AML (AUC = 0.8625, *P* < 0.0001). As early as in 2107, Li et al. (42) found that hsa_circ_0004277 was downregulated in AML and could serve as a diagnostic biomarker for AML. Subsequently, many circRNAs, including circ_ANAPC7 (43), circPAN3 (44), and circMYBL2 (30), were found to serve as the diagnostic biomarkers for AML. The results also showed that hsa_circ_0012152 could discriminate patients with AML from healthy individuals and that the circRNA expression profiles, hsa_circ_00001857, and hsa_circ_0012152 were promising potential classification biomarkers to discriminate ALL from AML providing an additional tool to the traditional tests used to categorize acute leukemia. Previous studies showed that the combinations of as few as two specifically expressed miRNAs could identify ALL from AML with an accuracy rate of >95% (45). Even the acute leukemia of ambiguous lineage could be defined as either ALL or AML using only five lineage-specific miRNAs (46). The lncRNA expression had also been shown to discriminate AL subtypes (47). Similarly, the results showed that combining hsa_circ_00001857 and hsa_circ_0012152 using the serial test, parallel test, or predictive model could more accurately discriminate ALL from AML.

A possible mechanism for the different expression patterns of hsa_circ_0001857 and hsa_circ_0012152 between ALL and AML might be that they participated in the leukemogenesis of ALL and AML, respectively. For example, circ-ANAPC7 participated in the occurrence and development of AML through the miR-181 family (43), circPAN3 mediated drug resistance through the miR-153-5p/miR-183-5p/XIAP axis (44), and circ-DLEU2 was involved in AML through the miR-496/PRKACB axis (48). The ceRNA network showed that hsa_circ_0012152 competed with EGFR and MAPK1 to combine miR-491-5p or miR-512-3p, and the GO and KEGG functional annotation showed that the target genes of hsa_circ_0012152 were involved in some biological processes such as “covalent chromatin modification,” “histone modification,” “Ras protein signal transduction,” and “myeloid cell differentiation” and might participate in such pathways as mitogen-activated protein kinase (MAPK) and phosphatidylinositol 3′-kinase (PI3K)-Akt signaling pathways. Furthermore, MAPK is an ubiquitous signaling molecule that regulates the proliferation and differentiation of various cells, including hematopoietic cells (49). It is continuously activated in patients with AML and cell lines, which is an independent poor prognostic factor for AML response to chemotherapy and overall survival (50, 51). The suppression of *MAPK* activation could inhibit the progression of AML cell cycle and promote the sensitivity of AML cells to chemotherapy (52). EGFR is an important therapeutic target for non-small cell lung cancer, and its binding to the ligand could induce dimerization and tyrosine residue autophosphorylation and activate the MAPK pathway (53). The EGFR expression pattern in AML is controversial; two patients with AML and non-small cell lung cancer achieved remission of AML after the use of EGFR inhibitors, indicating that EGFR was the potential therapeutic target of AML (54, 55). Mahmud H et al. (56) found that about 11% AML had higher EGFR expression than normal CD34+ cells in 511 AML patients, and 18% AML had high phosphorylated EGFR expression in 179 patients with AML. Sun et al. (57) found that EGFR was expressed in 33% patients with AML and indicated a poor outcome. Consequently, it was speculated that hsa_circ_0012152 might participate in the progression of AML by inhibiting the expression of miR-491-5p or miR-512-3p, promoting the expression of EGFR and MAPK1 and eventually activating the MAPK pathway. That is, hsa_circ_0012152 might involve in AML through miR-491-5p/EGFR/MAPK1 axis or the miR-512-3p/EGFR/MAPK1 axis. Importantly, this meant that, in addition to EGFR and MAPK as the therapeutic targets for AML, hsa_circ_0012152, miR-491-5p, and miR-512-3p might also be precise therapeutic targets for AML.

This study showed that hsa_circ_0012152, which was upregulated in AML, was a promising classification biomarker to discriminate ALL from AML and might participate in the progression of AML through the miR-491-5p/EGFR/MAPK1 axis or the miR-512-3p/EGFR/MAPK1 axis. Hsa_circ_0001857, which was upregulated in ALL, could serve as a classification biomarker to discriminate ALL from AML. The diagnostic efficiency would be improved by combining hsa_circ_0012152 and hsa_circ_0001857 for the serial test and logistic regression predictive model. The study provided an additional tool to improve the clinical management of AML, including diagnosis, classification, and targeted therapy. However, the results need to be verified using a larger sample size. In addition, the relationship between hsa_circ_0012152 or hsa_circ_0001857 and gene mutation, prognosis, and chemoresistance, as well as their mechanism of action in AL, remains to be further explored.

## Data Availability Statement

The raw data supporting the conclusions of this article will be made available by the authors, without undue reservation.

## Ethics Statement

The studies involving human participants were reviewed and approved by Ningbo First Hospital Ethics Committee. The patients/participants provided their written informed consent to participate in this study.

## Author Contributions

HH, GO, and QM were the principal investigators. SG and BL performed most of the experiments. YC, YZ, and NW performed part of the experiments. DZ and SY performed data analysis. SG wrote the manuscript. LS, HH, GO, and QM revised the manuscript. All authors contributed to the article and approved the submitted version.

## Conflict of Interest

The authors declare that the research was conducted in the absence of any commercial or financial relationships that could be construed as a potential conflict of interest.

## Footnotes

1 http://starbase.sysu.edu.cn/

2 http://string-db.org/

3 https://www.kegg.jp/
