# Supplementary material for: Hsa_circ_0012152 and Hsa_circ_0001857 Accurately Discriminate Acute Lymphoblastic Leukemia From Acute Myeloid Leukemia
Source: Front Oncol. 2020 Sep 2;10:1655. doi: 10.3389/fonc.2020.01655 (PMC7492294; doi:10.3389/fonc.2020.01655)
Supplement: Supplementary file 1 [file Data_Sheet_1.doc]

# Supplementary materials

## Table S1. Primers used for RT-qPCR

| CircRNAs/Gene | Primer Sequence (5＇→3＇) |
| --- | --- |
| GAPDH-F | ATGGGGAAGGTGAAGGTCG |
| GAPDH-R | GGGTCATTGATGGCAACAATATC |
| hsa_circ_0001857-F | TCCTCGGTGAGCACGGATTC |
| hsa_circ_0001857-R | ACAACCATGGCTGACCCGAA |
| hsa_circ_0001858-F | TACAGCAGCCACCCAACCAA |
| hsa_circ_0001858-R | CCCCAGGCTTGATGCTTCCT |
| hsa_circ_0074816-F | CATGCCATCCGTGTGCAGAC |
| hsa_circ_0074816-R | AGCATGGTACCGAATATGACCTGT |
| hsa_circ_0001681-F | TGTGCCTCTCATTCCTGCCA |
| hsa_circ_0001681-R | CCAACGCAGCTCCTCCTGTA |
| hsa_circ_0012152-F  hsa_circ_0012152-R | TCTCCCCACTTGCGCTTCTC  GCCAACCAGCACTTTGGGTC |

## Table S2. Top 10 differentially expressed circRNAs between ALL and AML

| CircRNA | log_2_FC | P-value | FDR | Dysregulation | | Gene Symbol | Location |
| --- | --- | --- | --- | --- | --- | --- | --- |
| hsa_circ_0020093 | 7.84 | 4.13E-06 | 0.003502 | up | | *ATRNL1* | Chr10 |
| hsa_circ_0001857 | 7.56 | 1.19E-05 | 0.005522 | up | | *PAX5* | Chr 9 |
| hsa_circ_0001858 | 7.08 | 1.54E-05 | 0.006610 | up | | *PAX5* | Chr 9 |
| hsa_circ_0074816 | 6.50 | 1.36E-06 | 0.001688 | | up | *EBF1* | Chr 5 |
| hsa_circ_0001681 | 5.56 | 1.09E-05 | 0.005259 | | up | *RAPGEF5* | Chr 7 |
| hsa_circ_0012152 | -6.28 | 4.56E-06 | 0.003569 | | down | *RNF220* | Chr1 |
| hsa_circ_0035197 | -4.92 | 3.10E-08 | 0.000158 | | down | *ATP8B4* | Chr15 |
| hsa_circ_0067997 | -4.89 | 2.07E-05 | 0.007248 | | down | *FNDC3B* | Chr3 |
| hsa_circ_0004087 | -4.00 | 8.03E-06 | 0.004545 | | down | *CDYL2* | Chr16 |
| hsa_circ_0001334 | -3.85 | 2.32E-05 | 0.007851 | | down | *MCM2* | Chr3 |

## Table S3: The diagnostic efficiency of hsa_circ_0001857 and hsa_circ_0012512 as diagnostic biomarkers

| CircRNA | Sensitivity | Specificity | AUC | 95% CI | *P* value |
| --- | --- | --- | --- | --- | --- |
| hsa_circ_0001857 | 0.950 | 0.600 | 0.7350 | 0.4456 – 1.0000 | 0.0896 |
| hsa_circ_0012152 | 0.977 | 1.000 | 0.9773 | 0.9332 – 1.0000 | 0.0005 |


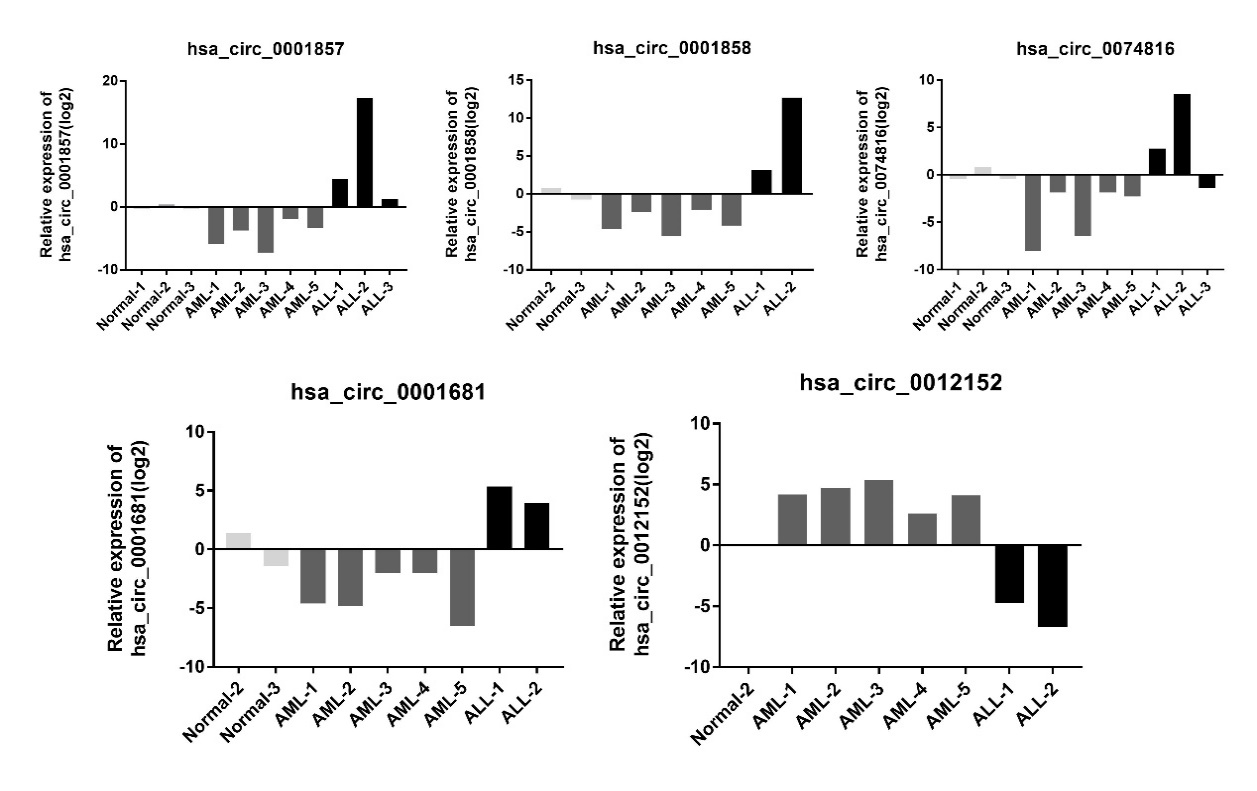


## Figure S1. Expression level of 5 circRNAs differentially expressed between ALL and AML. Due to the insufficient of samples Normal-1, Normal-3 and ALL-3, the expression levels of hsa_circ_0001858 and hsa_circ_0001681 in Normal-1 and ALL-3 were not quantified, and the expression levels of hsa_circ_0012152 in Normal-1, Normal-3 and ALL-3 were not quantified.

A B


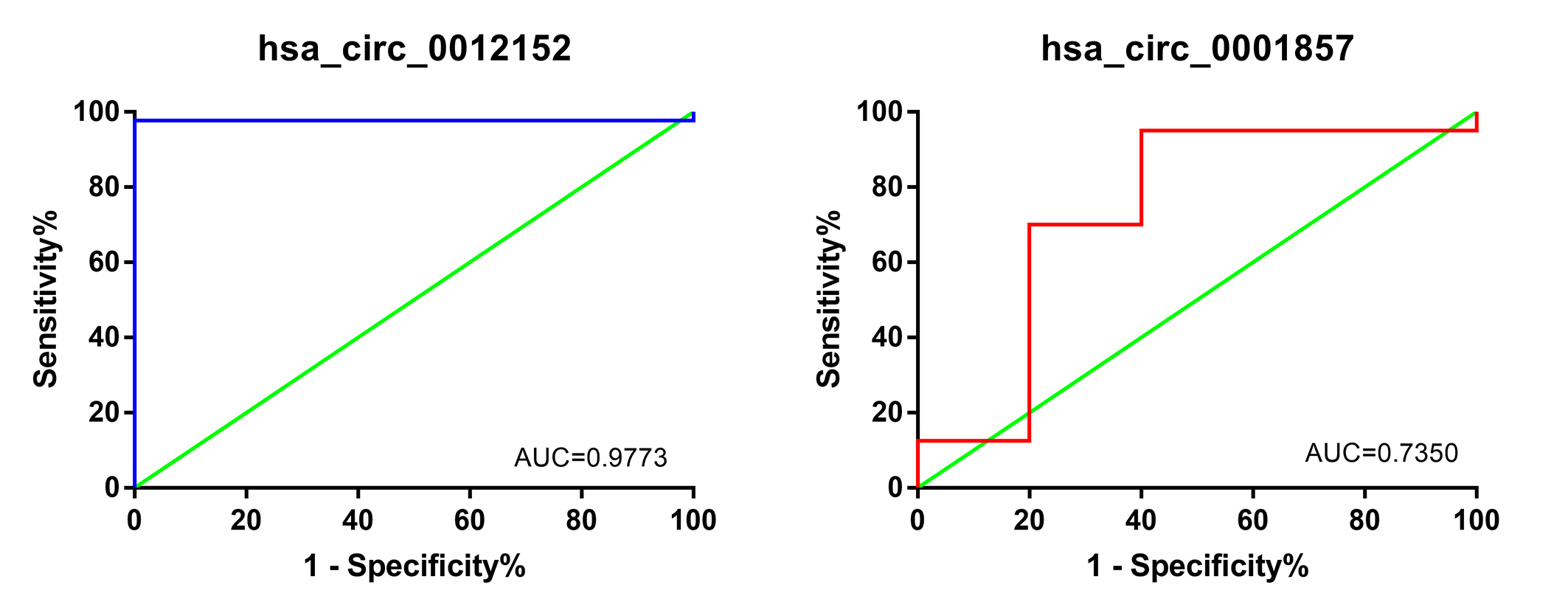


## Figure S2. The ROC of hsa_circ_00012152 and hsa_circ_0001857 as diagnosis biomarkers to discriminate AML or ALL from healthy individuals. (A) The ROC of hsa_circ_0012152 as a diagnostic biomarker to discriminate AML from the healthy individuals. (B) The ROC of hsa_circ_0001857 as a diagnostic biomarker to discriminate ALL from the heathy individuals.


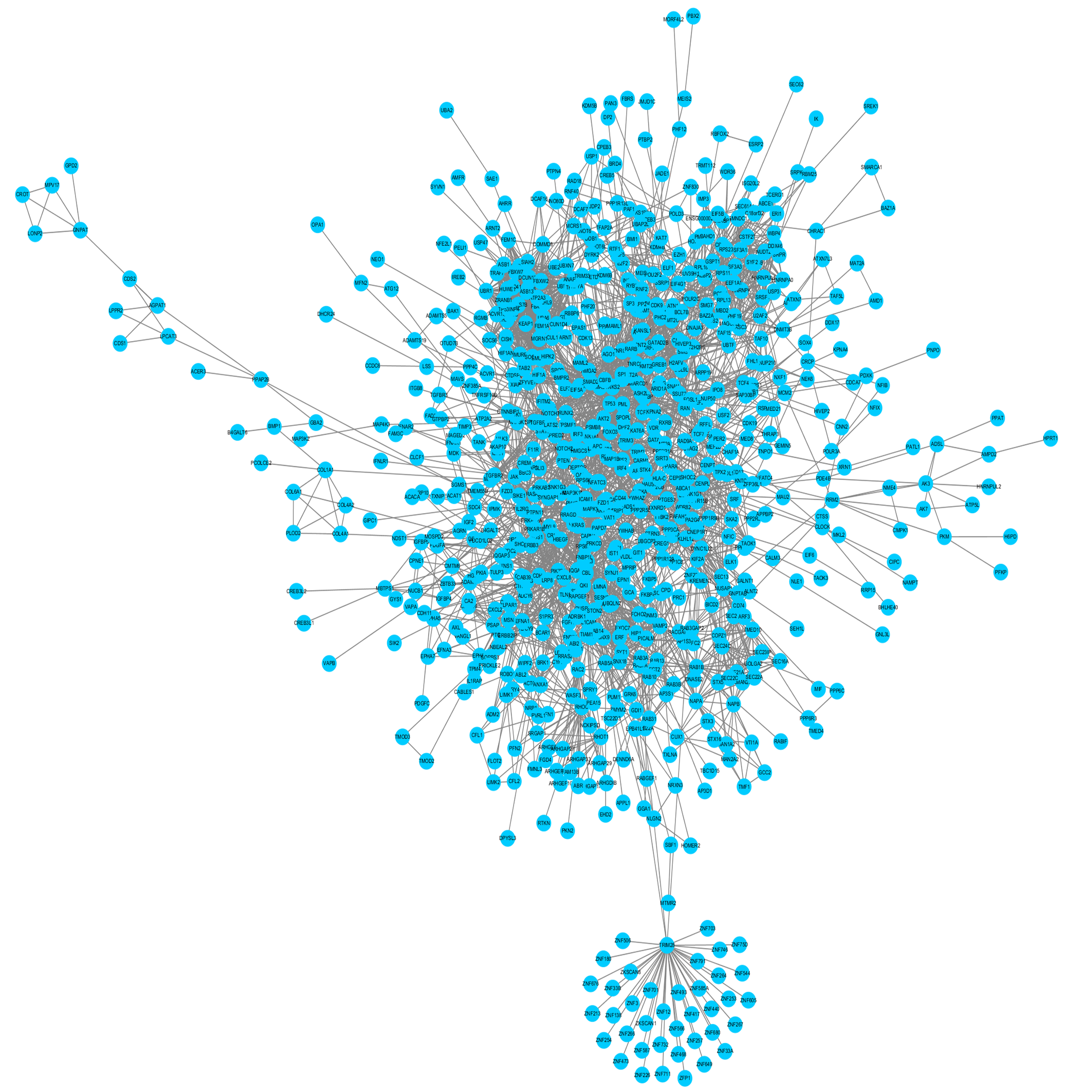


## Figure S3: PPI network of the target genes of hsa_circ_0012152. Each node represents a protein and the lines between the nodes show the interaction between proteins.


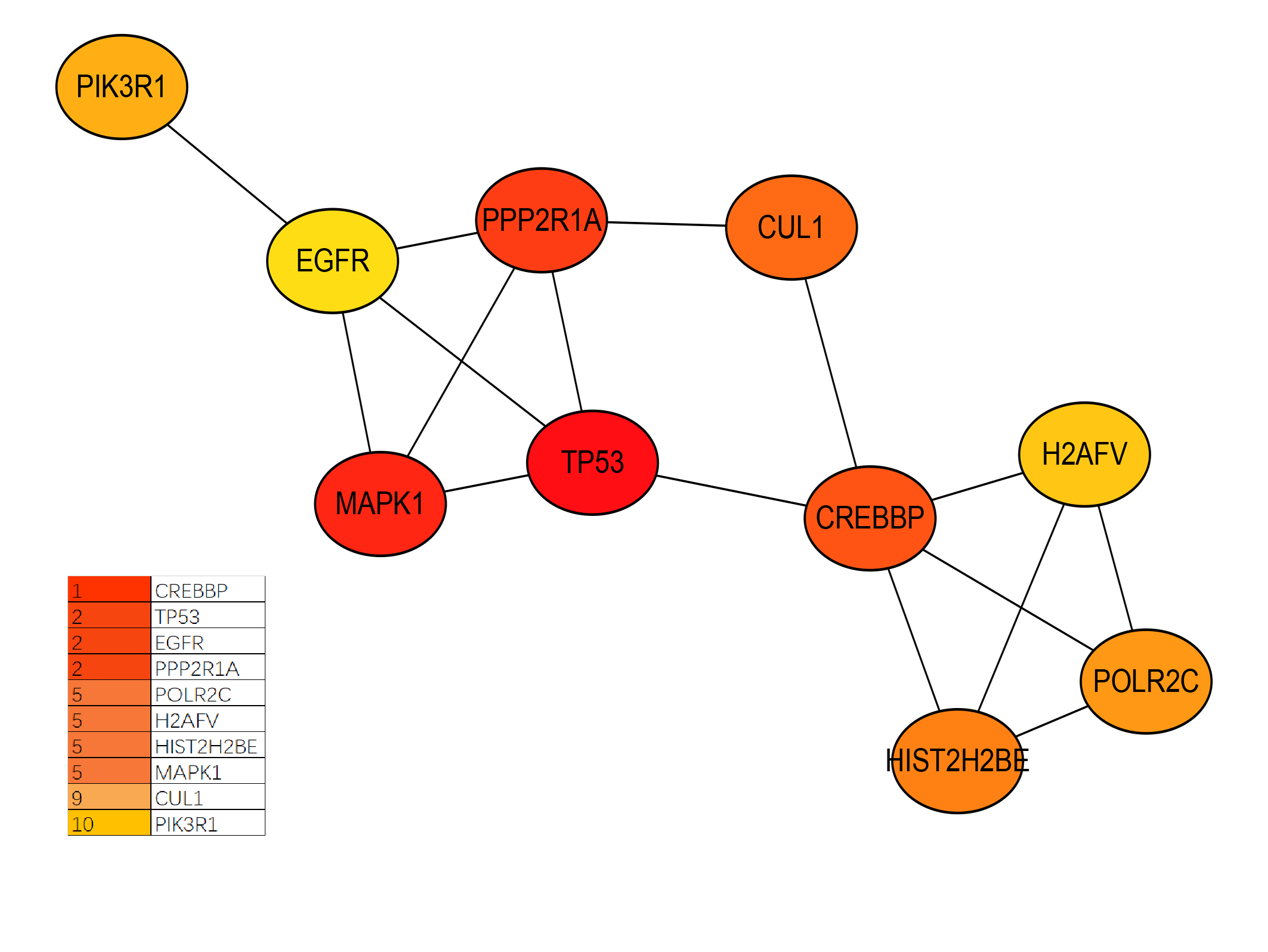


## Figure S4: 10 hub genes identified from the PPI network by cytoscape.


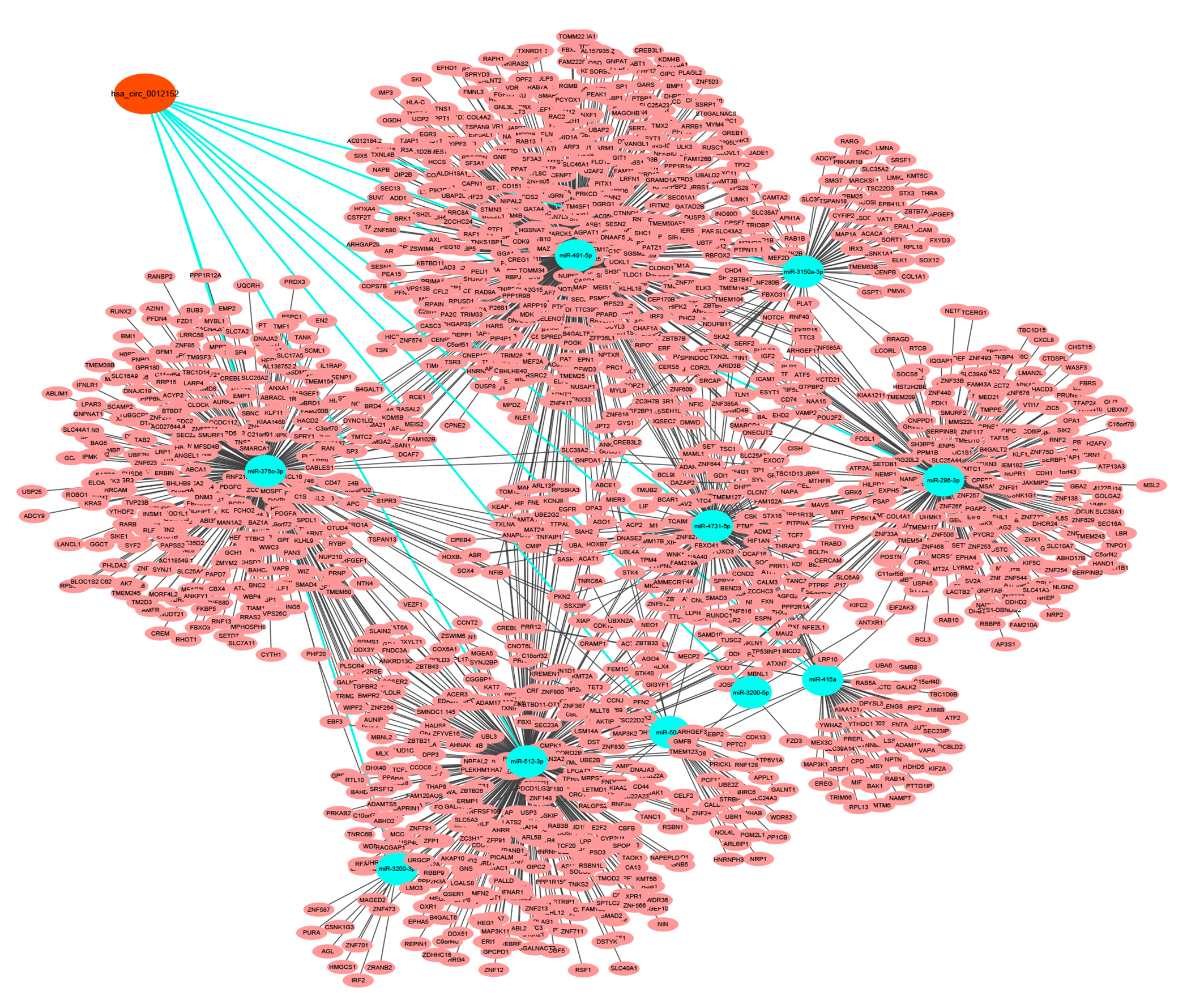


## Figure S5. The crosstalk network of circRNA-miRNA-mRNA about hsa_circ_0012152. The red node is hsa_circ_0012152, the blue nodes are miRNAs targeted by hsa_circ_0012152, the pink nodes are target genes of miRNAs, and the line between nodes show the interactions between circRNA and miRNAs or between miRNAs and target genes.
